# Influence of diabetes on survival of patients with glioma: a meta-analysis

**DOI:** 10.3389/fendo.2026.1667242

**Published:** 2026-02-12

**Authors:** Jing Yi, Jiangli Wen, Nianhua Wang, Min Yuan, Haibin Leng, Hua Chen

**Affiliations:** Department of Neurosurgery, Changde Hospital, Xiangya School of Medicine, Central South University (The First People’s Hospital of Changde City), Changde, Hunan, China

**Keywords:** diabetes, glioblastoma, glioma, progression, survival

## Abstract

**Background:**

The prognostic impact of preexisting diabetes on glioma survival remains unclear, with conflicting evidence across studies. This meta-analysis aimed to assess the association between diabetes and survival outcomes in patients with glioma.

**Methods:**

We systematically searched PubMed, Embase, and Web of Science up to April 30, 2025, for cohort studies reporting hazard ratios (HRs) for overall survival (OS) or progression-free survival (PFS) in glioma patients with and without preexisting diabetes. A random-effects model was used to pool multivariate-adjusted HRs accounting for the possible influence of heterogeneity.

**Results:**

Twelve retrospective cohort studies comprising 8,948 patients were included. Eleven studies reported on OS, and four on PFS. Pooled analysis showed that preexisting diabetes was associated with poorer OS (HR: 1.22, 95% CI: 1.10–1.36, p < 0.001; I² = 33%). Sensitivity analysis confirmed result robustness (HR range: 1.18–1.26). Subgroup analyses revealed consistent associations regardless of tumor grade (Grade III–IV: HR: 1.42; GBM: HR: 1.19; p for subgroup difference = 0.47), age (< 60 vs. ≥ 60 years, p = 0.15), sex distribution (< 60% vs. ≥ 60% men, p = 0.91), diabetes type (type 2 vs. overall diabetes, p = 0.33), or follow-up duration (≤ 12 vs. >12 months, p = 0.69). Preexisting diabetes was also associated with poorer PFS (HR: 1.36, 95% CI: 1.01–1.83, p = 0.04; I² = 0%).

**Conclusions:**

Preexisting diabetes is associated with reduced survival in glioma patients. These findings highlight the importance of integrating metabolic comorbidities into glioma prognostic assessment.

**Systematic Review Registration:**

https://www.crd.york.ac.uk/PROSPERO/view/CRD420251086248, identifier CRD420251086248.

## Introduction

Gliomas are the most common malignant primary brain tumors in adults, with glioblastoma (GBM) representing the most aggressive and lethal subtype (1, 2). Despite advancements in multimodal treatment—including maximal surgical resection followed by radiotherapy and temozolomide chemotherapy—the prognosis for glioma, particularly the high-grade glioma and GBM, remains dismal (3). The median overall survival of GBM is typically less than 15 months, and the 5-year survival rate is below 10% (4, 5). Even with optimal therapy, GBM is characterized by rapid progression, frequent recurrence, and marked resistance to conventional treatment (6, 7). Accordingly, identifying prognostic factors that influence survival is essential to refine risk stratification and guide individualized management (8, 9). In addition to tumor-related features, patient-level metabolic comorbidities have emerged as potential modifiers of cancer progression and outcomes (10).

Diabetes, a global health burden with increasing prevalence, has been linked to the prognosis of several malignancies, including pancreatic, breast, and colorectal cancers (11, 12). The mechanisms underlying the potential influence of diabetes on cancer survival are multifactorial and may involve hyperglycemia, insulin resistance, chronic inflammation, and altered cellular metabolism (13, 14). In glioma, preclinical studies suggest that hyperglycemia may promote tumor growth (15), while insulin and insulin-like growth factor signaling pathways may contribute to resistance to apoptosis and radiotherapy (16). However, clinical evidence on the association between diabetes and glioma survival remains inconsistent, with some studies reporting worse outcomes (17–19) and others showing null effects (20–28). To clarify this issue, we conducted a systematic review and meta-analysis to examine the influence of pre-existing diabetes on the overall survival (OS) and progression-free survival (PFS) of patients with glioma. By synthesizing available evidence, this study aims to quantify the prognostic impact of diabetes, explore potential sources of heterogeneity, and provide insights into whether diabetes status should be considered in glioma prognostic models and clinical decision-making.

## Methods

### Protocol and registration

This meta-analysis was conducted in accordance with the PRISMA 2020 (29) and Cochrane Handbook guidelines (30). The protocol was registered in the PROSPERO database (Registration number: CRD420251086248).

### Eligibility criteria

We applied the PICOS framework to define the inclusion criteria:

Population (P): Adult patients (≥ 18 years) with histologically confirmed glioma of any WHO grade.
Intervention/Exposure (I): Presence of pre-existing diabetes mellitus (type 1 or type 2), as defined by medical history, medical records, or laboratory parameters.
Comparison (C): Glioma patients without preexisting diabetes.
Outcomes (O): The primary outcome was OS, and the secondary outcomes included PFS if available. OS was usually defined as the time from treatment start to death from any cause. PFS was the time from treatment start to disease progression or death.
Study Design (S): Cohort studies (prospective or retrospective) reporting adjusted or unadjusted hazard ratios (HRs) and corresponding 95% confidence intervals (CIs) for the association between diabetes and the survival outcome of patients with glioma.

Exclusion criteria included reviews, editorials, case reports, meta-analyses, preclinical studies, cross-sectional studies, studies not reporting survival outcomes, or those where diabetes status could not be separated from other metabolic conditions. If studies had overlapping populations, we included the one with the largest sample size in the meta-analysis.

### Search strategy

A comprehensive search was performed in PubMed, Embase, and Web of Science from database inception to April 30, 2025. The search combined terms related to glioma and diabetes using both keywords and controlled vocabulary (e.g., MeSH and Emtree terms), which involved the combination of (1) “diabetes” OR “diabetic” OR “T1DM” OR “T2DM” OR “hyperglycemia”; (2) “glioma” OR “glioblastoma” OR “oligodendroglioma” OR “astrocytoma” OR “oligoastrocytoma” OR “ependymoma” OR “brain cancer” OR “cerebral cancer” OR “intracranial cancer”; and (3) “survival” OR “progression” OR “recurrence” OR “death” OR “mortality” OR “metastasis” OR “cohort” OR “longitudinal” OR “follow-up” OR “followed” OR “follow” OR “prospective” OR “retrospective” OR “prospectively” OR “retrospectively” OR “prognosis” OR “clinical outcome”. The search was restricted to studies on human subjects and included only full-length articles published in English in peer-reviewed journals. We also manually checked the references of related original and review articles to find additional relevant studies. The detailed search strategy for each database is shown in **Supplementary File 1**.

### Study selection and risk of bias evaluation

Two reviewers independently screened titles and abstracts, followed by full-text review to assess eligibility. Discrepancies were resolved by consensus or discussion with the corresponding author. We screened for duplicate/overlapping cohorts by cross-checking study setting(s), recruitment period, sample size, and author lists. When overlap was suspected, we retained the dataset with the largest sample, most recent accrual, and most complete covariate adjustment (and molecular/treatment detail where available). Companion papers were included only if they reported distinct outcomes or non-overlapping time windows. When non-overlap could not be verified, we selected a single primary report to avoid double counting. Two authors independently performed the literature search, study selection, quality assessment, and data extraction. Disagreements were also resolved by discussion with the corresponding author. Study quality was assessed using the Newcastle-Ottawa Scale (NOS) (31), which rates selection, control of confounders, and outcome evaluation. Scores range from 1 to 9, with scores of 7 or higher considered good quality.

### Data extraction

A standardized data extraction form was used to collect study characteristics (author, year, country), patient demographics, glioma grade, main anticancer treatment, diabetes definition and numbers of patients with preexisting diabetes, outcome measures (OS, PFS), follow-up duration, effect estimates (HRs with 95% confidence intervals), and adjustment covariates.

### Data synthesis and statistical analysis

We used HRs and 95% CIs to assess the association between preexisting diabetes and survival in patients with glioma, comparing between patients with and without preexisting diabetes. For each study we extracted all reported effect estimates and their covariate sets, then selected the maximally adjusted hazard ratio for pooling (pre-specified hierarchy: multivariable HR > stratified HR > unadjusted HR). HRs and standard errors were directly extracted or calculated from 95% CIs or p values, then log-transformed to stabilize variance and normalize the data (30). If multiple HRs were reported from different models, we used the one with the most complete adjustment. Heterogeneity was assessed using the Cochrane Q test and I² statistic (32), with a p value < 0.10 suggesting significant heterogeneity and I² values of < 25%, 25–75%, and > 75% indicating low, moderate, and high heterogeneity. A random-effects model was used to pool the data, accounting for heterogeneity between studies (30). For the primary outcome of OS, sensitivity analyses were done by removing one study at a time. Predefined subgroup analyses were conducted based on tumor grade, average patient age, sex distribution, type of preexisting diabetes, and mean follow-up durations. Medians of continuous variables were used to divide subgroups evenly. Publication bias was assessed using funnel plots and visual inspection for asymmetry, along with Egger’s test (33). All analyses were performed using RevMan (Version 5.1; Cochrane Collaboration, Oxford, UK) and Stata (Version 12.0; Stata Corporation, College Station, TX, USA).

## Results

### Study inclusion

**Figure 1:**
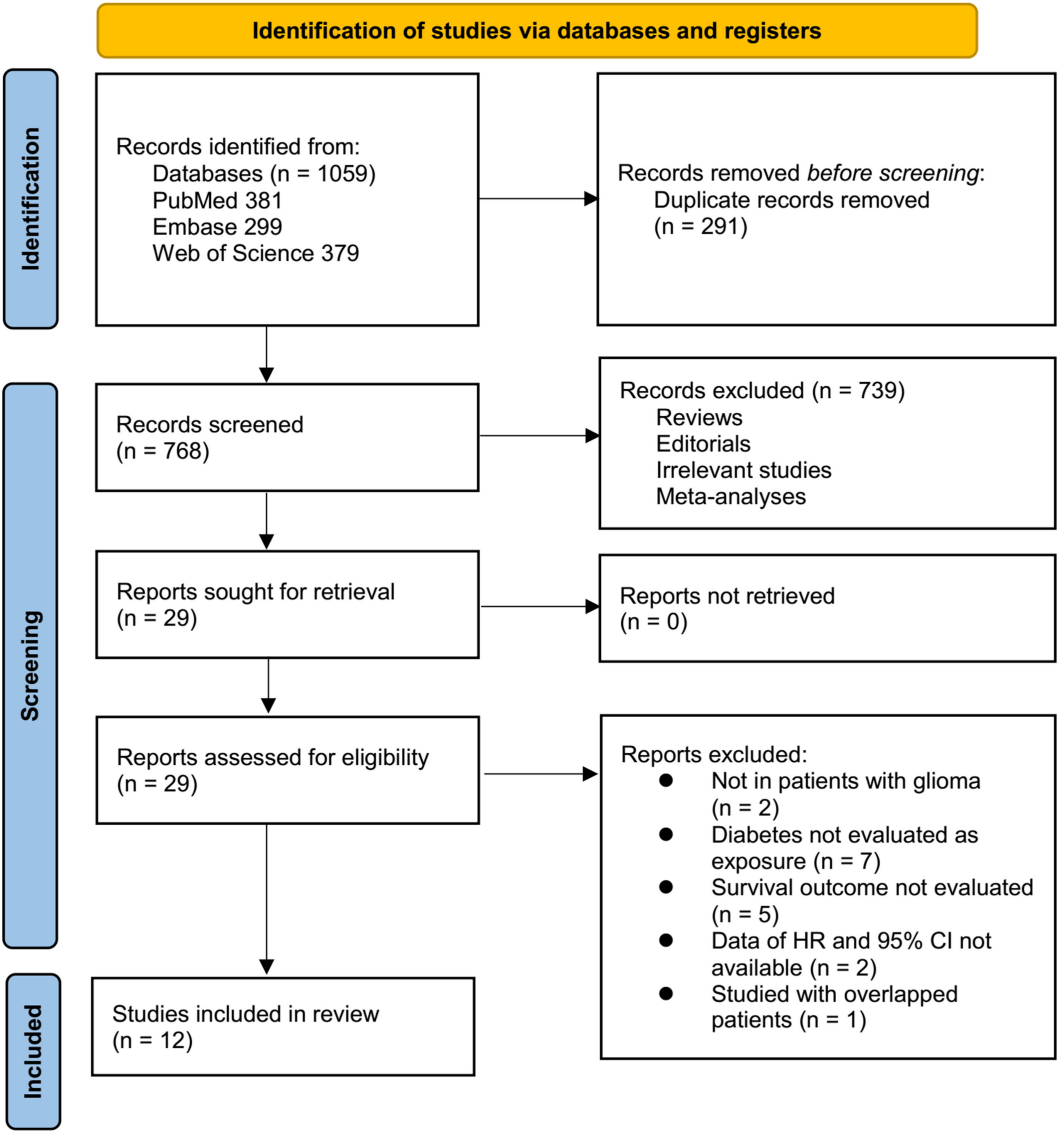
Flowchart of database search and study inclusion.

The study selection process is shown in **Figure 1**. We first identified 1,059 records from the three databases. After removing 291 duplicates, 768 articles were screened by title and abstract. Of these, 739 were excluded for not meeting the aims of the meta-analysis. The full texts of the remaining 29 articles were reviewed by two independent authors, and 17 were excluded for various reasons (see **Figure 1**). In the end, 12 studies were included in the quantitative analysis (17–28).

### Summary of study characteristics and study quality evaluation

**Table 1** summarizes the key characteristics of the 12 studies included in this meta-analysis, all of which were retrospective cohort designs published between 2010 and 2025 across various countries, including the United States, Canada, Germany, Turkey, India, and China. A total of 8,948 patients with high-grade glioma were included. Three of the studies included patients with grade III to IV glioma (17, 20, 21), while the other nine studies included only patients with GBM (18, 19, 22–28). Most included studies enrolled patients with glioblastoma at the time of initial diagnosis, whereas none were limited to recurrent cases. Three studies (19, 24, 28) explicitly analyzed newly diagnosed GBM cohorts treated with standard concurrent chemoradiotherapy. Molecular data were available in three studies: two (19, 28) restricted analyses to isocitrate dehydrogenase (IDH)-wild-type glioblastoma, while one (24) reported partial IDH-1 testing (~32% tested, 6% mutated). Earlier studies published before 2016 did not include molecular information, consistent with historical WHO classifications. In the study by Tieu et al. (24), dexamethasone exposure was recorded as a time-weighted mean and included in multivariable adjustment, reducing bias from steroid-induced hyperglycemia. Overall, the pooled evidence primarily represents newly diagnosed, predominantly IDH-wild-type GBM populations. Treatment descriptions in the included reports were heterogeneous and high-level; none explicitly documented compliance with the National Comprehensive Cancer Network (NCCN) or the European Association of Neuro-Oncology (EANO) guidance or adherence/completion metrics (e.g., dose intensity, number of TMZ cycles). Sample sizes varied widely, ranging from 60 to 3,784 participants, with mean ages spanning from 54.0 to 74.6 years and men comprising between 57% and 70% of study populations. Preexisting diabetes, predominantly type 2 (T2DM) in six studies (17, 22, 24, 25, 27, 28) and overall diabetes in the other six studies (18–21, 23, 26), was defined based on medical records or database codes. Overall, 1,337 (14.9%) of the included patients were with preexisting diabetes. The median follow-up duration ranged from 6 to 48.6 months. OS was reported in 11 studies (17–26, 28), followed by PFS in four studies (17, 19, 23, 27). Most studies adjusted for key prognostic variables, such as age, performance status, extent of surgery, and adjuvant therapies (17–26, 28), while one study reported no adjustments and only univariate data can be extracted (27). Study quality, assessed using the NOS (**Table 2**), showed total scores ranging from 6 to 9, indicating moderate to high methodological quality. The itemized NOS ratings and justifications for each domain and study are shown in **Supplementary Table 1**. Eleven studies scored ≥ 7 (17–26, 28), suggesting high quality, while only one study scored 6 (27), reflecting moderate quality due to incomplete adjustment for confounders and possible selective bias. All studies had adequate outcome assessment and follow-up completeness, supporting the reliability of survival data.

**Table 1:** Characteristics of the included studies.

Study	Country	Study design	Diagnosis	Main treatment	No. of patients	Mean age (years)	Men (%)	Definition of DM	No. of patients with DM	Median follow-up duration (months)	Outcomes	Variables adjusted
Grommes 2010 (20)	USA	RC	Glioma (grade III-IV)	Standard therapy (surgery, radiation, chemotherapy)	302	61	57	Preexisting DM, medical records validated	49	40	OS	Age, comorbidities, KPS, surgery type, histology, RPA class, tumor location, PPARγ status, radiation, gender
Chambless 2012 (17)	USA	RC	Glioma (grade III-IV)	Surgical resection	171	55	NR	Preexisting T2DM diagnosed by a physician via ADA criteria, validated via medical records	15	12	OS and PFS	Age, KPS, extent of resection, TMZ, radiation therapy, and BMI
Welch 2013 (22)	USA	RC	GBM	Surgical resection (biopsy, subtotal, or gross-total) + adjuvant therapy (chemotherapy, radiation)	988	66	70	Preexisting T2DM, validated via medical records	123	25	OS	Age, KPS, resection extent, steroid dependency, metformin/sulfonylurea use, chemotherapy/radiation
Siegel 2013 (21)	USA	RC	Glioma (grade III-IV)	Surgical resection and adjuvant therapy	853	57	59.3	Preexisting DM validated by medical records	123	19	OS	Age, sex, KPS, SOC treatment, and BMI
Tieu 2015 (24)	Canada	RC	GBM	Radiotherapy and TMZ	393	54	64	Preexisting T2DM, validated via medical records	36	14	OS	Age, ECOG performance status, extent of surgery, TWM dexamethasone dose, BMI
Adeberg 2015 (23)	Germany	RC	GBM	Radiotherapy and TMZ	276	63	61.2	Preexisting DM, validation via clinical records	40	42	OS and PFS	Age, KPS, MGMT status, resection status, TMZ therapy, and corticosteroid use
Chen 2017 (18)	USA	RC	GBM	Surgical resection	3784	74.6	62.5	Preexisting DM, validated by ICD codes	599	6	OS	Age, Gagne comorbidity score, sex, race, extent of surgery, smoking status, prior craniotomy
Barami 2017 (25)	USA	RC	GBM	Surgical resection ± adjuvant therapy	1144	63	57.4	Preexisting T2DM, validated by WHO criteria (HbA1c ≥6.5%)	168	12	OS	Age, race, sex, hyperlipidemia, obesity, year of diagnosis
Potharaju 2018 (26)	India	RC	GBM	Maximal safe resection + concurrent chemoradiation (TMZ + RT) + adjuvant temozolomide (6 cycles)	392	56	68.6	Preexisting clinically diagnosis of DM, ICD codes/medical records validated	105	48.6	OS	Age, KPS, extent of resection, RPA class, BMI category
Mohammad 2023 (28)	Canada	RC	GBM	Surgical resection/biopsy + adjuvant therapy (TMZ + RT)	241	64.5	58.9	Preexisting T2DM, validated via medical records	43	10.3	OS	Age, BMI, KPS, extent of resection, MGMT status, adjuvant therapy, dexamethasone use, infectious/thrombotic events
Kocaeli 2023 (27)	Turkey	RC	GBM	Surgical resection/biopsy + adjuvant therapy	60	60.4	68.3	Preexisting T2DM per ADA criteria. Validation: Clinical records and HbA1c levels.	13	42.5	PFS	None
Zha 2025 (19)	China	RC	GBM	Surgical resection + CCRT ± targeted therapy	344	54	58.7	Preexisting clinically diagnosis of DM, medical records validated	23	13.3	OS and PFS	Age, sex, hypertension, KPS, tumor size, resection extent, targeted therapy, and TMT

RC, retrospective cohort; GBM, glioblastoma; DM, diabetes mellitus; T2DM, type 2 diabetes mellitus; OS, overall survival; PFS, progression-free survival; KPS, Karnofsky performance status RPA, recursive partitioning analysis; PPARγ, peroxisome proliferator-activated receptor gamma; TMZ, temozolomide; SOC, standard of care; ECOG, Eastern Cooperative Oncology Group; MGMT, O6-methylguanine-DNA methyltransferase; ICD, International Classification of Diseases; HbA1c, glycated hemoglobin; RT, radiotherapy; TWM, time-weighted mean; CCRT, concurrent chemoradiotherapy; TMT, temozolomide therapy; NR, not reported.

**Table 2:** Characteristics of the included studies.

Study	Representativeness of the exposed cohort	Selection of the non exposed cohort	Ascertainment of exposure	Outcome not present at baseline	Control for age	Control for other confounding factors	Assessment of outcome	Enough long follow-up duration	Adequacy of follow-up of cohorts	Total
Grommes 2010 (20)	0	1	1	1	1	1	1	1	1	8
Chambless 2012 (17)	0	1	1	1	1	1	1	1	1	8
Welch 2013 (22)	0	1	1	1	1	1	1	1	1	8
Siegel 2013 (21)	1	1	1	1	1	1	1	1	1	9
Tieu 2015 (24)	0	1	1	1	1	1	1	1	1	8
Adeberg 2015 (23)	1	1	1	1	1	1	1	1	1	9
Chen 2017 (18)	0	1	1	1	1	1	1	0	1	7
Barami 2017 (25)	0	1	1	1	1	1	1	1	1	8
Potharaju 2018 (26)	0	1	1	1	1	1	1	1	1	8
Mohammad 2023 (28)	0	1	1	1	1	1	1	0	1	7
Kocaeli 2023 (27)	0	1	1	1	0	0	1	1	1	6
Zha 2025 (19)	1	1	1	1	1	1	1	1	1	9

### Association between diabetes and OS

**Figure 2:**
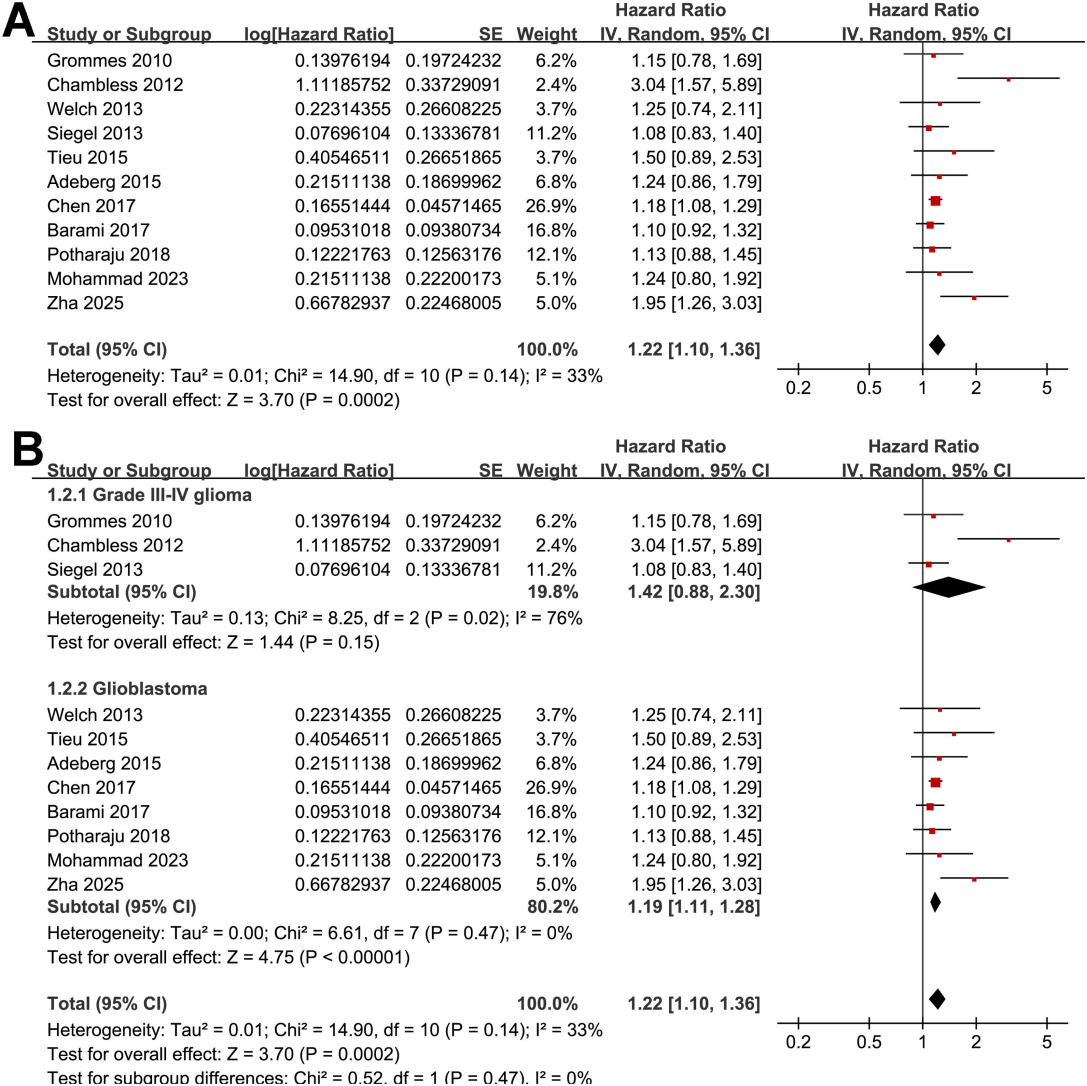
Forest plots for the meta-analysis of the association between preexisting diabetes and OS of patients with glioma; **(A)** overall meta-analysis; and **(B)** subgroup analysis according to tumor grade.

**Figure 3:**
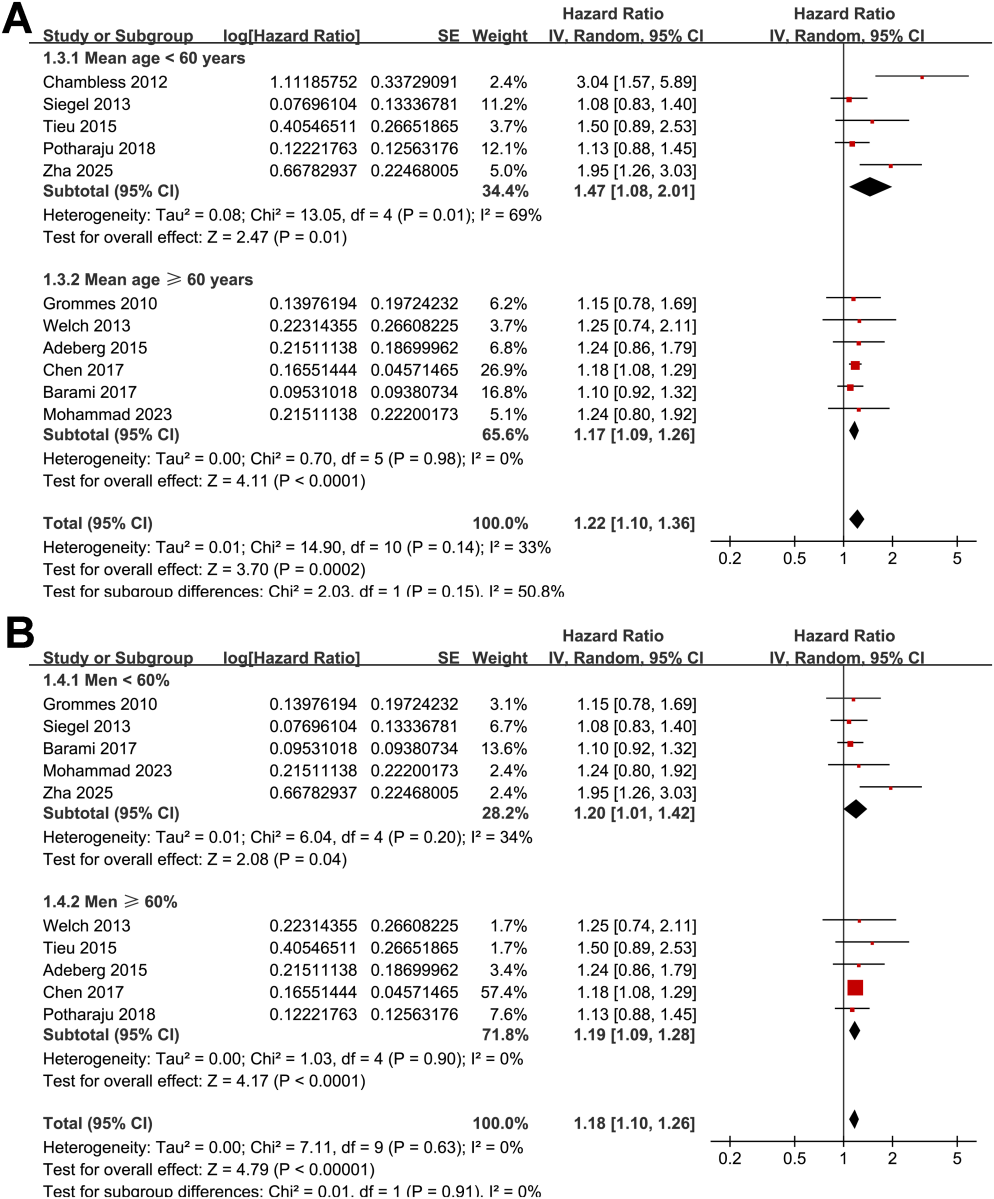
Forest plots for the subgroup analyses of the association between preexisting diabetes and OS of patients with glioma; **(A)** subgroup analysis according to the mean ages of the patients; and **(B)** subgroup analysis according to the proportion of men in each study.

**Figure 4:**
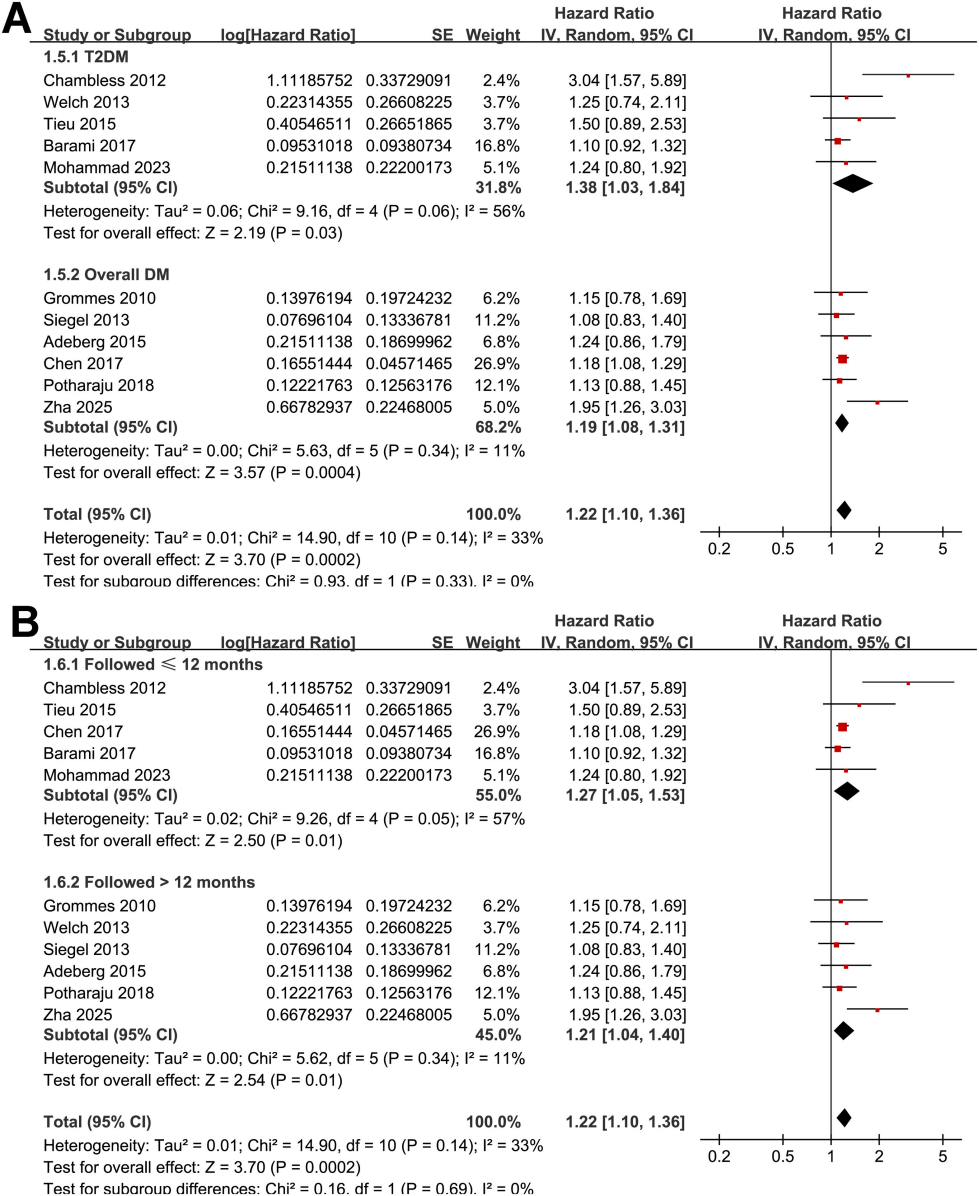
Forest plots for the subgroup analyses of the association between preexisting diabetes and OS of patients with glioma; **(A)** subgroup analysis according to the type of diabetes; and **(B)** subgroup analysis according to the follow-up durations.

A total of 11 cohorts (17–26, 28), all with the multivariate-adjusted data and NOS ≥ 7, reported the association between preexisting of diabetes and OS in patients with high grade glioma. Moderate heterogeneity was observed (p for Cochrane Q test = 0.14; I^2^ = 33%). Pooled results with a random-effects model showed that overall, preexisting diabetes was associated with poor OS (HR: 1.22, 95% CI: 1.10 to 1.36, p < 0.001; **Figure 2A**). Sensitivity analyses were performed by removing one dataset at a time, and the results remained stable (HR: 1.18–1.26, p all < 0.05). Further subgroup analyses indicated that the association between preexisting diabetes and poor OS was consistent in studies involving grade III-IV glioma and in studies involving patients with GBM only (HR: 1.42 vs. 1.19, p for subgroup difference = 0.47; **Figure 2B**), in patients with mean ages < or ≥ 60 years (HR: 1.47 vs. 1.17, p for subgroup difference = 0.15; **Figure 3A**), between patient populations with the proportion of men < or ≥ 60% (HR: 1.20 vs. 1.19, p for subgroup difference = 0.91; **Figure 3B**), in studies evaluating preexisting T2DM or overall diabetes (HR: 1.38 vs. 1.19, p for subgroup difference = 0.33; **Figure 4A**), and in studies with follow-up durations ≤ or > 12 months (HR: 1.27 vs. 1.21, *p* for subgroup difference = 0.69; **Figure 4B**).

### Association between diabetes and PFS

**Figure 5:**
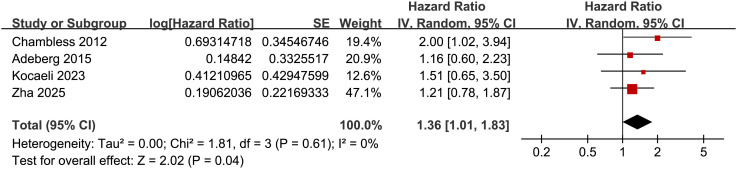
Forest plots for the meta-analysis of the association between preexisting diabetes and PFS of patients with glioma.

Further meta-analysis involving four cohorts (17, 19, 23, 27) showed that preexisting diabetes was also associated with poor PFS in patients with glioma (HR: 1.36, 95% CI: 1.01 to 1.83, p = 0.04; **Figure 5**) with no statistically significant heterogeneity (p for Cochrane Q test = 0.61; I^2^ = 0%). The results becomes non-significant if the only study with NOS = 6 and univariate analysis (27) was excluded (R: 1.34, 95% CI: 0.97 to 1.84, p = 0.07; I^2^ = 0%).

### Publication bias

**Figure 6:**
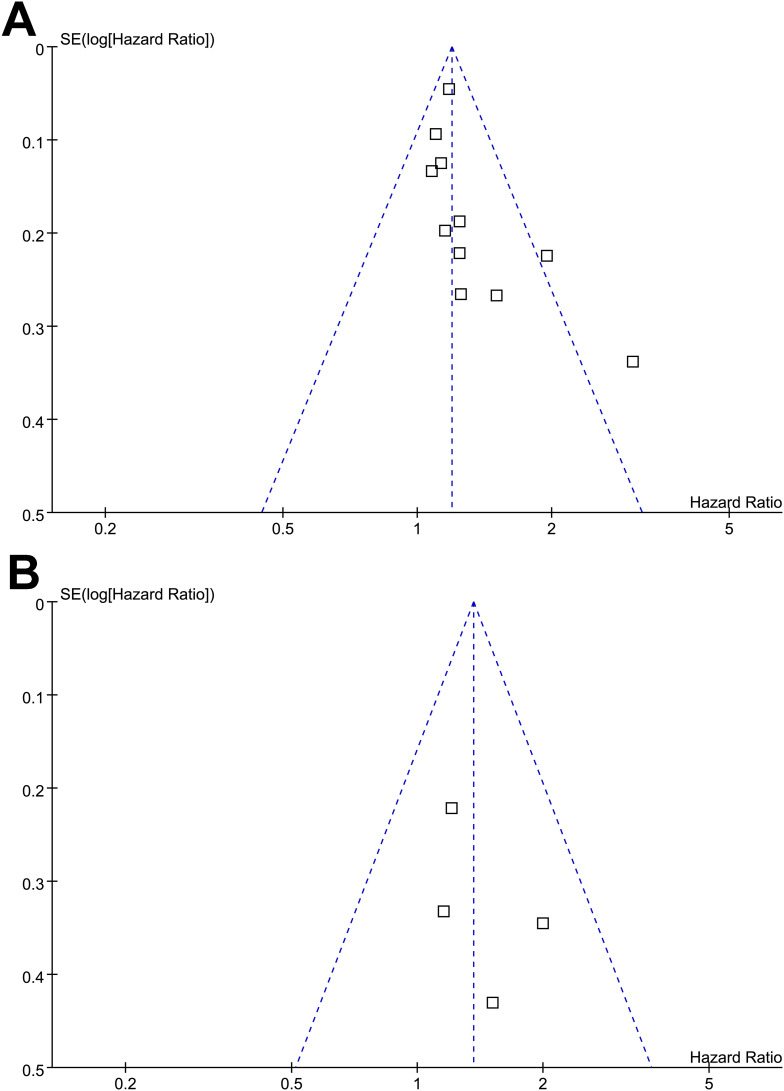
Funnel plots for estimating the potential publication biases underlying the meta-analyses of the association between preexisting diabetes and survival outcomes of patients with glioma; **(A)** funnel plots for the meta-analysis of the association between preexisting diabetes and OS; and **(B)** funnel plots for the meta-analysis of the association between preexisting diabetes and PFS.

Funnel plots for the meta-analyses of preexisting diabetes and survival outcomes in patients with glioma are shown in **Figures 6A, B**. The plots appeared symmetrical, suggesting a low risk of publication bias. For the outcome of OS, Egger’s test also showed no evidence of publication bias (*p* = 0.22). For the outcome of PFS, Egger’s test was not performed because only four studies were included.

## Discussion

This meta-analysis provides a comprehensive synthesis of current evidence on the association between pre-existing diabetes mellitus and survival in patients with high-grade glioma. Pooling data from 12 retrospective cohort studies encompassing nearly 8,500 patients, we found that pre-existing diabetes is significantly associated with reduced OS and, to a lesser extent, PFS. These findings indicate that diabetes is a clinically relevant comorbidity that may influence glioma prognosis beyond established tumor- and treatment-related factors.

Several biological and clinical mechanisms may underlie the observed associations between diabetes and adverse glioma outcomes. At the molecular level, chronic hyperglycemia is known to promote cancer cell proliferation, inhibit apoptosis, and enhance resistance to therapy through multiple pathways (34, 35). Elevated glucose levels fuel anaerobic glycolysis in glioma cells, supporting tumor growth and metabolic adaptation—a phenomenon known as the Warburg effect (36, 37). Hyperinsulinemia and insulin resistance, hallmarks of type 2 diabetes, may further activate insulin and insulin-like growth factor (IGF) signaling pathways, stimulating downstream cascades such as PI3K/AKT/mTOR, which are involved in glioma proliferation, angiogenesis, and treatment resistance (38, 39). Additionally, diabetes is a pro-inflammatory state, characterized by elevated levels of cytokines and reactive oxygen species, which may exacerbate glioma aggressiveness and disrupt the tumor immune microenvironment (40). In glioma specifically, preclinical studies have shown that hyperglycemia may accelerate tumor growth by upregulating the expression of the receptor for advanced glycation end products and suppressing antitumor immune responses (41). Moreover, hyperglycemia may actively drive glioblastoma progression by inducing cellular alterations in the tumor microenvironment and activating signaling pathways associated with glioblastoma development (42). From a clinical standpoint, diabetic patients may be more susceptible to treatment-related complications, including poor wound healing, infections, and corticosteroid-induced metabolic disturbances, which could compromise the effectiveness of surgery, radiotherapy, or chemotherapy (43, 44). Future studies are warranted to determine the key molecular pathways underlying the association between diabetes and poor prognosis of patients with high-grade glioma.

The subgroup analyses conducted in this study offer further insights into the robustness and potential modifiers of the association between diabetes and glioma outcomes. The association between preexisting diabetes and poor OS remained consistent across subgroups defined by glioma grade (grade III–IV vs. GBM-only), age, sex distribution, type of diabetes definition (type 2 diabetes vs. overall diabetes), and follow-up duration. Notably, the association appeared stronger in studies including grade III–IV gliomas compared to GBM-only cohorts, although this difference was not statistically significant. This may reflect the possibly more pronounced impact of comorbid conditions in lower-grade tumors with relatively longer survival, whereas in GBM, the rapid disease course may mask some of the effects of systemic factors like diabetes (9). Similarly, the association was slightly stronger in younger patients and those with shorter follow-up durations, which could suggest an early impact of metabolic dysregulation on tumor progression (45). Nonetheless, none of the subgroup differences reached statistical significance, supporting the generalizability of the findings across patient and study characteristics.

The association between pre-existing diabetes and poorer survival in high-grade glioma likely reflects a combination of biological and care-delivery factors. Outcomes in glioma depend on tumor grade and molecular profile, performance status, extent of resection, and delivery of guideline-concordant chemoradiation; however, several cohorts lacked granular molecular data and treatment-adherence details, leaving room for residual confounding. Most studies captured diabetes as a single, pre-existing category without separating prediabetes or newly diagnosed diabetes at admission, and few quantified corticosteroid exposure or stress hyperglycaemia. Such non-differential misclassification (undiagnosed dysglycemia in the comparison group) would be expected to bias estimates toward the null, suggesting the observed effect may be conservative. Future work should stratify by molecular markers and treatment intensity and incorporate standardized measures of glycemic status (HbA1c, peri-treatment glucose) and steroid dosing.

This study has several methodological strengths. First, all included studies were longitudinal cohort designs, which are more appropriate than cross-sectional studies for examining prognostic associations. Second, the meta-analysis focused on multivariate-adjusted hazard ratios for OS, enhancing the credibility of the findings by accounting for key clinical confounders such as age, performance status, extent of resection, and adjuvant treatment. Finally, a series of prespecified subgroup analyses and sensitivity analyses were performed, all of which confirmed the stability of the main findings.

Nonetheless, several limitations should be considered when interpreting the results. First, recurrence status was inconsistently reported, and survival outcomes were rarely stratified by newly diagnosed versus recurrent glioma. Although most cohorts explicitly or implicitly represented newly diagnosed cases, potential differences in treatment history or steroid exposure cannot be entirely excluded. Second, molecular markers such as IDH mutation or O6-methylguanine-DNA methyltransferase (MGMT) methylation were incompletely reported—only three studies provided IDH information, and two restricted analysis to IDH-wild-type tumors. As tumor molecular subtype substantially affects prognosis, the absence of such data in earlier cohorts may contribute to residual heterogeneity and limits molecular-level interpretation of our pooled estimates. Moreover, all included studies were retrospective, making the analyses vulnerable to recall bias, incomplete or inconsistently recorded clinical data, and unmeasured confounding (46). Key factors such as steroid exposure, treatment intensity, glycemic control, and molecular features were variably reported, and such omissions may have biased the observed associations in either direction. Although pooled associations reached statistical significance, the effect sizes were modest (HR = 1.22). In the context of high-grade glioma—where survival is short and shaped by multiple competing determinants—even modest relative increases in mortality risk may carry clinical relevance, especially at a population level. Moreover, such attenuated estimates are likely conservative given potential non-differential misclassification of dysglycemia and residual confounding related to steroid exposure or treatment intensity. In addition, residual confounding (e.g., performance status, extent of resection, molecular profile, treatment intensity, steroid exposure, stress hyperglycaemia) and selective reporting remain plausible. Therefore, these findings should be interpreted as hypothesis-generating rather than causal, and their clinical relevance should be weighed against the modest magnitude and between-study variability. While the studies adjusted for several clinical variables, residual confounding from unmeasured or inaccurately captured factors cannot be excluded. Also, because all included cohorts involved GBM or grade III–IV tumors, the findings primarily apply to high-grade disease. The prognostic role of diabetes in lower-grade (WHO I–II) gliomas remains uncertain, and our results should not be generalized to these populations without dedicated studies designed to evaluate this question. Moreover, although diabetes was generally identified from medical records in these retrospective cohorts and in several studies further validated using ADA criteria or HbA1c thresholds, heterogeneity in diagnostic approaches may have introduced some non-differential misclassification. However, the consistency of results across subgroup analyses comparing T2DM–specific versus overall diabetes definitions suggests that variation in ascertainment methods was unlikely to materially affect the pooled estimates. Furthermore, diabetes was treated as a binary baseline variable in all cohorts. Longitudinal glycemic control, diabetes duration, and treatment changes were rarely reported, and only isolated studies provided limited parameters such as HbA1c or steroid-adjusted glucose. The absence of such time-varying metabolic data precluded assessment of dose–response patterns and likely contributed to residual confounding. Prospective studies incorporating dynamic glycemic measures and detailed diabetes management are therefore warranted. Also, these factors may themselves influence glioma progression and survival, potentially confounding the observed associations. For example, the use of metformin has been associated with improved survival in several cancers, including glioma, and may attenuate the adverse impact of diabetes (47). In addition, we could not separate prediabetes or newly diagnosed diabetes from established diabetes because such strata were not reported. This may introduce non-differential misclassification (e.g., undiagnosed dysglycemia in the reference group), likely attenuating associations and contributing to heterogeneity. Future studies should prospectively distinguish prediabetes, newly diagnosed, and established diabetes, document steroid use and stress hyperglycemia, and evaluate dose–response by glycemic category. Besides, treatment delivery details were inconsistently reported across studies, and no article specified NCCN/EANO compliance or adherence/completion rates. This lack of detail may contribute to residual confounding related to treatment intensity and quality of care. Additionally, the analysis was based on aggregate study-level data rather than individual patient data, precluding more nuanced adjustment and subgrouping. Moreover, although preexisting diabetes was associated with poorer PFS in the pooled analysis, this finding is based on only four studies, limiting assessment of publication bias and overall certainty. The borderline significance and attenuation to non-significance after excluding the single unadjusted, lower-quality study suggest that the PFS association should be viewed as exploratory rather than conclusive. Finally, given the observational nature of the included studies, a causal relationship between diabetes and glioma outcomes cannot be definitively established.

From a clinical perspective, these findings support the consideration of diabetes as a potential adverse prognostic factor in patients with high-grade glioma. While tumor biology remains the dominant determinant of outcome, patient-level comorbidities such as diabetes may further modulate survival trajectories and should not be overlooked. Routine assessment of glycemic status and optimization of metabolic control may be important components of holistic glioma care. Moreover, future studies are warranted to explore whether tighter glycemic control or the use of specific antidiabetic agents, such as metformin, could improve outcomes in this patient population. Prospective studies with individual-level data collection on metabolic parameters, treatment regimens, and inflammatory markers will be essential to elucidate the mechanistic pathways linking diabetes to glioma progression and to identify modifiable targets for intervention. Pending definitive evidence, diabetes status should be routinely documented in neuro-oncology care, with baseline HbA1c and peri-treatment glucose monitoring and steroid-sparing strategies where clinically feasible. Although data on antidiabetic therapies, particularly metformin, were limited and inconsistently reported, their potential to modulate insulin/IGF signaling and tumor metabolism warrants prospective evaluation. Well-designed studies should distinguish prediabetes, newly diagnosed, and established diabetes; predefine glycemic targets; systematically record steroid exposure and stress hyperglycaemia; and assess whether metabolic optimization and specific antidiabetic agents improve survival or treatment tolerance—ideally in molecularly annotated cohorts.

## Conclusions

In conclusion, this meta-analysis indicates that pre-existing diabetes is associated with poorer OS and PFS in patients with high-grade glioma. However, the effect sizes are modest and the evidence is entirely retrospective, leaving room for residual confounding. These findings support routine documentation of diabetes in neuro-oncology care and cautious consideration of diabetes status in prognostic modeling where validated. Prospective, molecularly annotated studies with standardized glycemic metrics, explicit reporting of steroid exposure and stress hyperglycaemia, and careful accounting of treatment intensity are needed to establish independence of effect and to test whether metabolic optimization can meaningfully improve outcomes.

## Data Availability

The original contributions presented in the study are included in the article/**Supplementary Material**. Further inquiries can be directed to the corresponding authors.
