# Supplementary material for: Influence of diabetes on survival of patients with glioma: a meta-analysis
Source: Front Endocrinol (Lausanne). 2026 Feb 12;17:1667242. doi: 10.3389/fendo.2026.1667242 (PMC12935661; doi:10.3389/fendo.2026.1667242)
Supplement: Supplementary file 1 [file Supplementaryfile1.docx]

**Detailed search strategy for each database**

**PubMed**

#1 "Diabetes Mellitus"[Mesh] OR diabetes[tiab] OR diabetic[tiab] OR T1DM[tiab] OR T2DM[tiab] OR hyperglycemia[tiab]

#2 "Glioma"[Mesh] OR glioma[tiab] OR glioblastoma[tiab] OR oligodendroglioma[tiab] OR astrocytoma[tiab] OR oligoastrocytoma[tiab] OR ependymoma[tiab] OR "brain cancer"[tiab] OR "cerebral cancer"[tiab] OR "intracranial cancer"[tiab]

#3 "Survival"[Mesh] OR "Disease Progression"[Mesh] OR survival[tiab] OR progression[tiab] OR recurrence[tiab] OR death[tiab] OR mortality[tiab] OR metastasis[tiab] OR cohort[tiab] OR longitudinal[tiab] OR follow-up[tiab] OR followed[tiab] OR follow[tiab] OR prospective[tiab] OR retrospective[tiab] OR prospectively[tiab] OR retrospectively[tiab] OR prognosis[tiab] OR "clinical outcome"[tiab]

#4 #1 AND #2 AND #3

**Embase**

#1 'diabetes mellitus'/exp OR diabetes:ti,ab OR diabetic:ti,ab OR T1DM:ti,ab OR T2DM:ti,ab OR hyperglycemia:ti,ab

#2 'glioma'/exp OR glioma:ti,ab OR glioblastoma:ti,ab OR oligodendroglioma:ti,ab OR astrocytoma:ti,ab OR oligoastrocytoma:ti,ab OR ependymoma:ti,ab OR 'brain cancer':ti,ab OR 'cerebral cancer':ti,ab OR 'intracranial cancer':ti,ab

#3 'survival'/exp OR 'disease progression'/exp OR survival:ti,ab OR progression:ti,ab OR recurrence:ti,ab OR death:ti,ab OR mortality:ti,ab OR metastasis:ti,ab OR cohort:ti,ab OR longitudinal:ti,ab OR 'follow up':ti,ab OR followed:ti,ab OR follow:ti,ab OR prospective:ti,ab OR retrospective:ti,ab OR prospectively:ti,ab OR retrospectively:ti,ab OR prognosis:ti,ab OR 'clinical outcome':ti,ab

#4 #1 AND #2 AND #3

**Web of Science**

TS=(diabetes OR diabetic OR T1DM OR T2DM OR hyperglycemia)

AND

TS=(glioma OR glioblastoma OR oligodendroglioma OR astrocytoma OR oligoastrocytoma OR ependymoma OR "brain cancer" OR "cerebral cancer" OR "intracranial cancer")

AND

TS=(survival OR progression OR recurrence OR death OR mortality OR metastasis OR cohort OR longitudinal OR "follow-up" OR followed OR follow OR prospective OR retrospective OR prospectively OR retrospectively OR prognosis OR "clinical outcome")
