# Supplementary material for: Influence of diabetes on survival of patients with glioma: a meta-analysis
Source: Front Endocrinol (Lausanne). 2026 Feb 12;17:1667242. doi: 10.3389/fendo.2026.1667242 (PMC12935661; doi:10.3389/fendo.2026.1667242)
Supplement: Supplementary file 2 [file Table1.docx]

**Supplementary Table 1 Itemized NOS (1/0) with rationale**

This table reproduces the per-item NOS points used in Table 2 for each included study (S1–S4 Selection; C1–C2 Comparability; O1–O3 Outcome), with a brief rationale for each 1/0 assignment. Totals equal Table 2.

## Grommes 2010

| Item | Point (1/0) | Rationale | Note |
| --- | --- | --- | --- |
| S1 Representativeness of the exposed cohort | 0 | exposed cohort representativeness not clearly consecutive/random |  |
| S2 Selection of the non-exposed cohort (same community) | 1 | comparators drawn from same hospital/registry population |  |
| S3 Ascertainment of exposure (secure record/structured) | 1 | exposure/comorbidity obtained from medical/registry records |  |
| S4 Outcome not present at baseline | 1 | survival outcome absent at baseline by design |  |
| C1 Control for age/sex (design or analysis) | 1 | age/sex adjusted or matched in analysis |  |
| C2 Control for other confounders (e.g., KPS, resection, steroids/MGMT) | 1 | adjusted for other prognostic factors (e.g., KPS, resection, treatment, steroids/MGMT) |  |
| O1 Assessment of outcome (independent/record linkage) | 1 | OS/PFS obtained via records/independent sources/imaging |  |
| O2 Follow-up duration adequate (≥12 months for OS or sufficient for outcome) | 1 | follow-up window reported as ≥12 months or sufficient for OS |  |
| O3 Adequacy of follow-up (complete or losses ≤20% and described) | 1 | loss to follow-up low (≤20%) or described as minimal |  |

Total NOS points: 8

## Chambless 2012

| Item | Point (1/0) | Rationale | Note |
| --- | --- | --- | --- |
| S1 Representativeness of the exposed cohort | 0 | exposed cohort representativeness not clearly consecutive/random |  |
| S2 Selection of the non-exposed cohort (same community) | 1 | comparators drawn from same hospital/registry population |  |
| S3 Ascertainment of exposure (secure record/structured) | 1 | exposure/comorbidity obtained from medical/registry records |  |
| S4 Outcome not present at baseline | 1 | survival outcome absent at baseline by design |  |
| C1 Control for age/sex (design or analysis) | 1 | age/sex adjusted or matched in analysis |  |
| C2 Control for other confounders (e.g., KPS, resection, steroids/MGMT) | 1 | adjusted for other prognostic factors (e.g., KPS, resection, treatment, steroids/MGMT) |  |
| O1 Assessment of outcome (independent/record linkage) | 1 | OS/PFS obtained via records/independent sources/imaging |  |
| O2 Follow-up duration adequate (≥12 months for OS or sufficient for outcome) | 1 | follow-up window reported as ≥12 months or sufficient for OS |  |
| O3 Adequacy of follow-up (complete or losses ≤20% and described) | 1 | loss to follow-up low (≤20%) or described as minimal |  |

Total NOS points: 8

## Welch 2013

| Item | Point (1/0) | Rationale | Note |
| --- | --- | --- | --- |
| S1 Representativeness of the exposed cohort | 0 | exposed cohort representativeness not clearly consecutive/random |  |
| S2 Selection of the non-exposed cohort (same community) | 1 | comparators drawn from same hospital/registry population |  |
| S3 Ascertainment of exposure (secure record/structured) | 1 | exposure/comorbidity obtained from medical/registry records |  |
| S4 Outcome not present at baseline | 1 | survival outcome absent at baseline by design |  |
| C1 Control for age/sex (design or analysis) | 1 | age/sex adjusted or matched in analysis |  |
| C2 Control for other confounders (e.g., KPS, resection, steroids/MGMT) | 1 | adjusted for other prognostic factors (e.g., KPS, resection, treatment, steroids/MGMT) |  |
| O1 Assessment of outcome (independent/record linkage) | 1 | OS/PFS obtained via records/independent sources/imaging |  |
| O2 Follow-up duration adequate (≥12 months for OS or sufficient for outcome) | 1 | follow-up window reported as ≥12 months or sufficient for OS |  |
| O3 Adequacy of follow-up (complete or losses ≤20% and described) | 1 | loss to follow-up low (≤20%) or described as minimal |  |

Total NOS points: 8

## Siegel 2013

| Item | Point (1/0) | Rationale | Note |
| --- | --- | --- | --- |
| S1 Representativeness of the exposed cohort | 1 | institutional/registry cohort; likely consecutive or population-based |  |
| S2 Selection of the non-exposed cohort (same community) | 1 | comparators drawn from same hospital/registry population |  |
| S3 Ascertainment of exposure (secure record/structured) | 1 | exposure/comorbidity obtained from medical/registry records |  |
| S4 Outcome not present at baseline | 1 | survival outcome absent at baseline by design |  |
| C1 Control for age/sex (design or analysis) | 1 | age/sex adjusted or matched in analysis |  |
| C2 Control for other confounders (e.g., KPS, resection, steroids/MGMT) | 1 | adjusted for other prognostic factors (e.g., KPS, resection, treatment, steroids/MGMT) |  |
| O1 Assessment of outcome (independent/record linkage) | 1 | OS/PFS obtained via records/independent sources/imaging |  |
| O2 Follow-up duration adequate (≥12 months for OS or sufficient for outcome) | 1 | follow-up window reported as ≥12 months or sufficient for OS |  |
| O3 Adequacy of follow-up (complete or losses ≤20% and described) | 1 | loss to follow-up low (≤20%) or described as minimal |  |

Total NOS points: 9

## Tieu 2015

| Item | Point (1/0) | Rationale | Note |
| --- | --- | --- | --- |
| S1 Representativeness of the exposed cohort | 0 | exposed cohort representativeness not clearly consecutive/random |  |
| S2 Selection of the non-exposed cohort (same community) | 1 | comparators drawn from same hospital/registry population |  |
| S3 Ascertainment of exposure (secure record/structured) | 1 | exposure/comorbidity obtained from medical/registry records |  |
| S4 Outcome not present at baseline | 1 | survival outcome absent at baseline by design |  |
| C1 Control for age/sex (design or analysis) | 1 | age/sex adjusted or matched in analysis |  |
| C2 Control for other confounders (e.g., KPS, resection, steroids/MGMT) | 1 | adjusted for other prognostic factors (e.g., KPS, resection, treatment, steroids/MGMT) |  |
| O1 Assessment of outcome (independent/record linkage) | 1 | OS/PFS obtained via records/independent sources/imaging |  |
| O2 Follow-up duration adequate (≥12 months for OS or sufficient for outcome) | 1 | follow-up window reported as ≥12 months or sufficient for OS |  |
| O3 Adequacy of follow-up (complete or losses ≤20% and described) | 1 | loss to follow-up low (≤20%) or described as minimal |  |

Total NOS points: 8

## Adeberg 2015

| Item | Point (1/0) | Rationale | Note |
| --- | --- | --- | --- |
| S1 Representativeness of the exposed cohort | 1 | institutional/registry cohort; likely consecutive or population-based |  |
| S2 Selection of the non-exposed cohort (same community) | 1 | comparators drawn from same hospital/registry population |  |
| S3 Ascertainment of exposure (secure record/structured) | 1 | exposure/comorbidity obtained from medical/registry records |  |
| S4 Outcome not present at baseline | 1 | survival outcome absent at baseline by design |  |
| C1 Control for age/sex (design or analysis) | 1 | age/sex adjusted or matched in analysis |  |
| C2 Control for other confounders (e.g., KPS, resection, steroids/MGMT) | 1 | adjusted for other prognostic factors (e.g., KPS, resection, treatment, steroids/MGMT) |  |
| O1 Assessment of outcome (independent/record linkage) | 1 | OS/PFS obtained via records/independent sources/imaging |  |
| O2 Follow-up duration adequate (≥12 months for OS or sufficient for outcome) | 1 | follow-up window reported as ≥12 months or sufficient for OS |  |
| O3 Adequacy of follow-up (complete or losses ≤20% and described) | 1 | loss to follow-up low (≤20%) or described as minimal |  |

Total NOS points: 9

## Chen 2017

| Item | Point (1/0) | Rationale | Note |
| --- | --- | --- | --- |
| S1 Representativeness of the exposed cohort | 0 | exposed cohort representativeness not clearly consecutive/random |  |
| S2 Selection of the non-exposed cohort (same community) | 1 | comparators drawn from same hospital/registry population |  |
| S3 Ascertainment of exposure (secure record/structured) | 1 | exposure/comorbidity obtained from medical/registry records |  |
| S4 Outcome not present at baseline | 1 | survival outcome absent at baseline by design |  |
| C1 Control for age/sex (design or analysis) | 1 | age/sex adjusted or matched in analysis |  |
| C2 Control for other confounders (e.g., KPS, resection, steroids/MGMT) | 1 | adjusted for other prognostic factors (e.g., KPS, resection, treatment, steroids/MGMT) |  |
| O1 Assessment of outcome (independent/record linkage) | 1 | OS/PFS obtained via records/independent sources/imaging |  |
| O2 Follow-up duration adequate (≥12 months for OS or sufficient for outcome) | 0 | follow-up length not clearly ≥12 months/sufficient |  |
| O3 Adequacy of follow-up (complete or losses ≤20% and described) | 1 | loss to follow-up low (≤20%) or described as minimal |  |

Total NOS points: 7

## Barami 2017

| Item | Point (1/0) | Rationale | Note |
| --- | --- | --- | --- |
| S1 Representativeness of the exposed cohort | 0 | exposed cohort representativeness not clearly consecutive/random |  |
| S2 Selection of the non-exposed cohort (same community) | 1 | comparators drawn from same hospital/registry population |  |
| S3 Ascertainment of exposure (secure record/structured) | 1 | exposure/comorbidity obtained from medical/registry records |  |
| S4 Outcome not present at baseline | 1 | survival outcome absent at baseline by design |  |
| C1 Control for age/sex (design or analysis) | 1 | age/sex adjusted or matched in analysis |  |
| C2 Control for other confounders (e.g., KPS, resection, steroids/MGMT) | 1 | adjusted for other prognostic factors (e.g., KPS, resection, treatment, steroids/MGMT) |  |
| O1 Assessment of outcome (independent/record linkage) | 1 | OS/PFS obtained via records/independent sources/imaging |  |
| O2 Follow-up duration adequate (≥12 months for OS or sufficient for outcome) | 1 | follow-up window reported as ≥12 months or sufficient for OS |  |
| O3 Adequacy of follow-up (complete or losses ≤20% and described) | 1 | loss to follow-up low (≤20%) or described as minimal |  |

Total NOS points: 8

## Potharaju 2018

| Item | Point (1/0) | Rationale | Note |
| --- | --- | --- | --- |
| S1 Representativeness of the exposed cohort | 0 | exposed cohort representativeness not clearly consecutive/random |  |
| S2 Selection of the non-exposed cohort (same community) | 1 | comparators drawn from same hospital/registry population |  |
| S3 Ascertainment of exposure (secure record/structured) | 1 | exposure/comorbidity obtained from medical/registry records |  |
| S4 Outcome not present at baseline | 1 | survival outcome absent at baseline by design |  |
| C1 Control for age/sex (design or analysis) | 1 | age/sex adjusted or matched in analysis |  |
| C2 Control for other confounders (e.g., KPS, resection, steroids/MGMT) | 1 | adjusted for other prognostic factors (e.g., KPS, resection, treatment, steroids/MGMT) |  |
| O1 Assessment of outcome (independent/record linkage) | 1 | OS/PFS obtained via records/independent sources/imaging |  |
| O2 Follow-up duration adequate (≥12 months for OS or sufficient for outcome) | 1 | follow-up window reported as ≥12 months or sufficient for OS |  |
| O3 Adequacy of follow-up (complete or losses ≤20% and described) | 1 | loss to follow-up low (≤20%) or described as minimal |  |

Total NOS points: 8

## Mohammad 2023

| Item | Point (1/0) | Rationale | Note |
| --- | --- | --- | --- |
| S1 Representativeness of the exposed cohort | 0 | exposed cohort representativeness not clearly consecutive/random |  |
| S2 Selection of the non-exposed cohort (same community) | 1 | comparators drawn from same hospital/registry population |  |
| S3 Ascertainment of exposure (secure record/structured) | 1 | exposure/comorbidity obtained from medical/registry records |  |
| S4 Outcome not present at baseline | 1 | survival outcome absent at baseline by design |  |
| C1 Control for age/sex (design or analysis) | 1 | age/sex adjusted or matched in analysis |  |
| C2 Control for other confounders (e.g., KPS, resection, steroids/MGMT) | 1 | adjusted for other prognostic factors (e.g., KPS, resection, treatment, steroids/MGMT) |  |
| O1 Assessment of outcome (independent/record linkage) | 1 | OS/PFS obtained via records/independent sources/imaging |  |
| O2 Follow-up duration adequate (≥12 months for OS or sufficient for outcome) | 0 | follow-up length not clearly ≥12 months/sufficient |  |
| O3 Adequacy of follow-up (complete or losses ≤20% and described) | 1 | loss to follow-up low (≤20%) or described as minimal |  |

Total NOS points: 7

## Kocaeli 2023

| Item | Point (1/0) | Rationale | Note |
| --- | --- | --- | --- |
| S1 Representativeness of the exposed cohort | 0 | exposed cohort representativeness not clearly consecutive/random |  |
| S2 Selection of the non-exposed cohort (same community) | 1 | comparators drawn from same hospital/registry population |  |
| S3 Ascertainment of exposure (secure record/structured) | 1 | exposure/comorbidity obtained from medical/registry records |  |
| S4 Outcome not present at baseline | 1 | survival outcome absent at baseline by design |  |
| C1 Control for age/sex (design or analysis) | 0 | no explicit adjustment/matching for age/sex |  |
| C2 Control for other confounders (e.g., KPS, resection, steroids/MGMT) | 0 | limited adjustment beyond age/sex |  |
| O1 Assessment of outcome (independent/record linkage) | 1 | OS/PFS obtained via records/independent sources/imaging |  |
| O2 Follow-up duration adequate (≥12 months for OS or sufficient for outcome) | 1 | follow-up window reported as ≥12 months or sufficient for OS |  |
| O3 Adequacy of follow-up (complete or losses ≤20% and described) | 1 | loss to follow-up low (≤20%) or described as minimal |  |

Total NOS points: 6

## Zha 2025

| Item | Point (1/0) | Rationale | Note |
| --- | --- | --- | --- |
| S1 Representativeness of the exposed cohort | 1 | institutional/registry cohort; likely consecutive or population-based |  |
| S2 Selection of the non-exposed cohort (same community) | 1 | comparators drawn from same hospital/registry population |  |
| S3 Ascertainment of exposure (secure record/structured) | 1 | exposure/comorbidity obtained from medical/registry records |  |
| S4 Outcome not present at baseline | 1 | survival outcome absent at baseline by design |  |
| C1 Control for age/sex (design or analysis) | 1 | age/sex adjusted or matched in analysis |  |
| C2 Control for other confounders (e.g., KPS, resection, steroids/MGMT) | 1 | adjusted for other prognostic factors (e.g., KPS, resection, treatment, steroids/MGMT) |  |
| O1 Assessment of outcome (independent/record linkage) | 1 | OS/PFS obtained via records/independent sources/imaging |  |
| O2 Follow-up duration adequate (≥12 months for OS or sufficient for outcome) | 1 | follow-up window reported as ≥12 months or sufficient for OS |  |
| O3 Adequacy of follow-up (complete or losses ≤20% and described) | 1 | loss to follow-up low (≤20%) or described as minimal |  |

Total NOS points: 9

Abbreviations: NOS = Newcastle–Ottawa Scale; OS = overall survival; PFS = progression-free survival; KPS = Karnofsky performance status; MGMT = O6-methylguanine-DNA methyltransferase.
